# Supplementary material for: Word contexts enhance the neural representation of individual letters in early visual cortex
Source: Nat Commun. 2020 Jan 16;11:321. doi: 10.1038/s41467-019-13996-4 (PMC6965097; doi:10.1038/s41467-019-13996-4)
Supplement: Supplementary file 1 — Supplementary Information [file 41467_2019_13996_MOESM1_ESM.pdf]

Supplementary information

Word contexts enhance the neural representation of individual letters in early visual cortex

Heilbron et al.

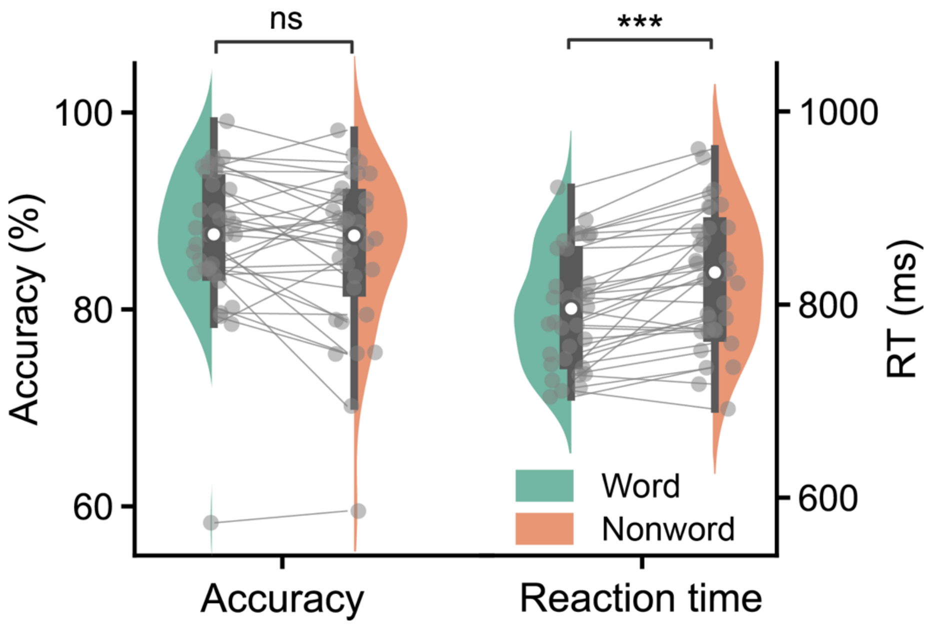

Supplementary Figure 1 Behavioural results. To make sure participants kept reading and were equally attentive of words and nonwords, they performed a challenging orthographic discrimination task. The task was performed on specific, learned targets that were presented about once per trial at an unpredictable moment. Targets were learned during a separate training session and were presented either in their regular (learned) form or with one of the non-middle letters permuted. Whenever a target was presented participants had to report whether it was correctly 'spelled'. Participants were faster (Wilcoxon signed rank, T=40, $p=1.07\times10^{-5}$, r = 0.87) but not statistically significantly more accurate (two-tailed t-test, t_{34} = 1.70, p = 0.098, d = 0.29) for word compared to nonword targets. This is in line with word superiority, although the perceptual nature of this advantage cannot be established from behavioural results on this task alone as there might also be memory or decisional factors contributing to the observed facilitation. Grey dots with connecting lines are individual participants. Colours are estimated densities, white dots are group medians, boxes are quartiles and whiskers are 1.5 interquartile range. Significance stars indicate p<0.001 (***) in a (paired) two-tailed Wilcoxon sign rank test.

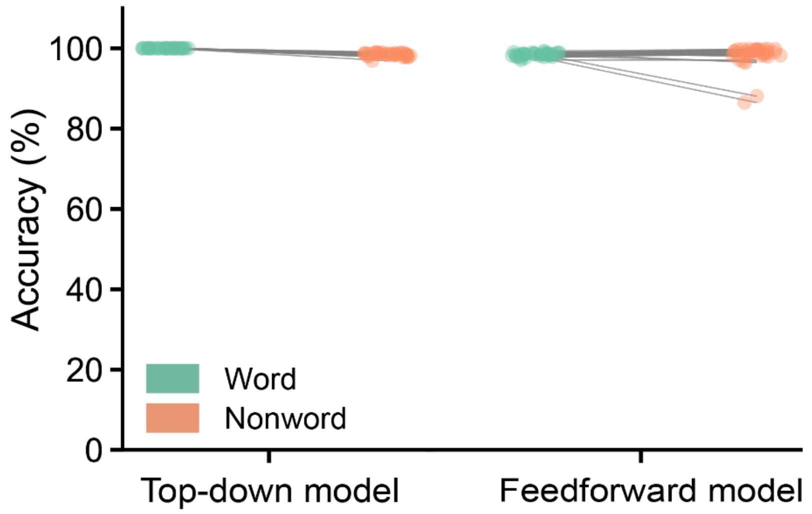

Supplementary Figure 2 Simulated letter identification accuracies. All simulation parameters were identical to the simulation of Figure 3a, except that median predicted response accuracy, rather than representational strength, for the middle letter was computed (see *Methods*). The fact that the accuracies are virtually at 100% in all conditions shows that stimuli were, despite the visual noise, clearly 'visible' to the network (note that chance level would be 3.84% or 1/26). This reflects a key difference between our paradigm – in which stimuli were presented well-above threshold – and the majority of studies in the literature – where stimuli are presented near-threshold. These results confirm that even when the critical letter is clearly visible and predicted letter identification responses are virtually at 100%, theoretical models still predict that enhancement of representations can occur. The accuracy values here might appear in conflict with the accuracies in Supplementary Figure 1. Note however that in the behavioural task, performance did not purely rely on perception of letters but also on their comparison to a memory template, and that the task was performed on the outer letters while participants maintained fixation at the centre of the screen. The middle letter was therefore always well-identifiable, making the predicted near-perfect accuracies a reasonable approximation of experimental viewing conditions.

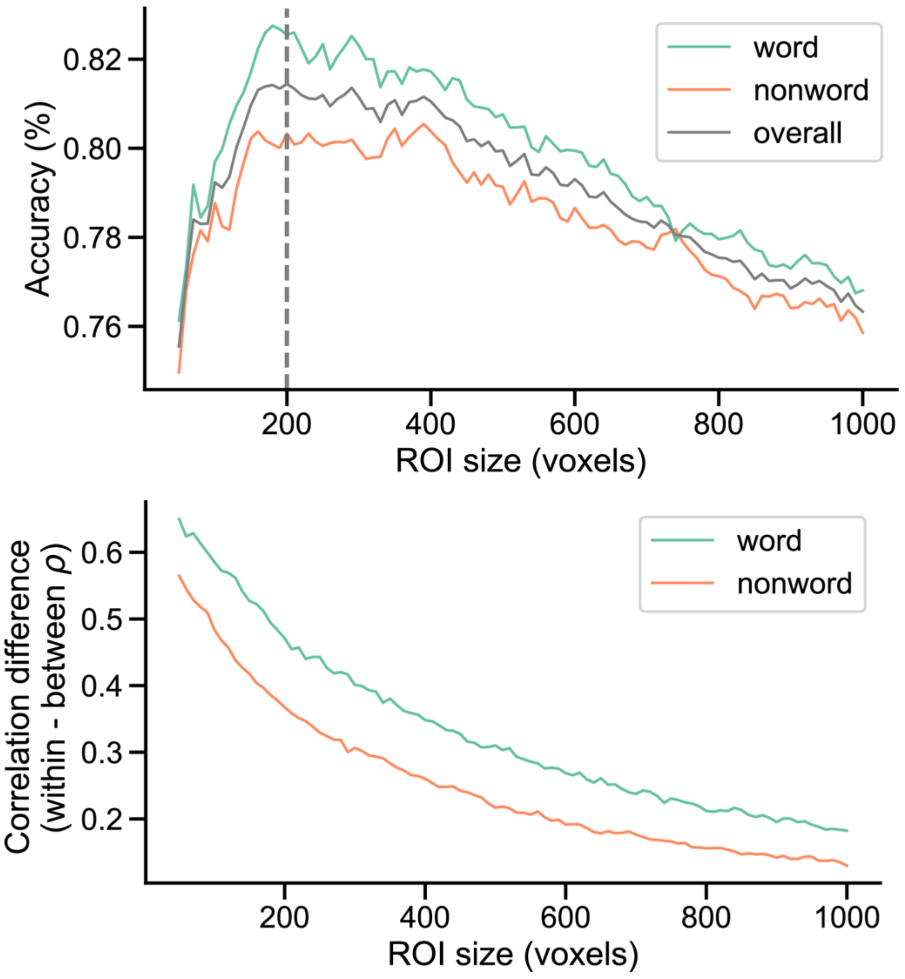

Supplementary Figure 3 Key contrast in main region of interest is stable over a range of ROI sizes. Same analysis as in Figure 3b, but performed over a wide range of ROI sizes, from 50 to 1000 voxels, with steps of 10. For both classification accuracy (upper panel) and pattern correlation difference (lower panel), the same pattern of effects was found practically over the full range of ROIs. Strikingly, the highest overall classification accuracy (vertical dashed line, corresponding to the maximum value of the solid grey line) was found at the pre-defined ROI of 200 voxels – a number that we based on a previous study[1]. Although the difference with other, similar ROI sizes is negligible, this result confirms that the choice for 200 voxels was justified in the sense that choosing a different number could not have considerably improved the decoding performance.

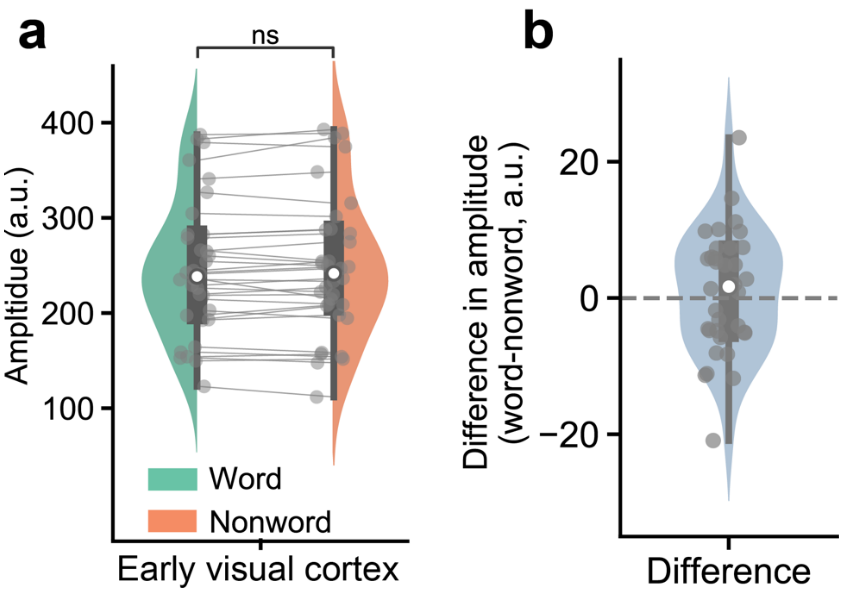

Supplementary Figure 4 No difference in amplitude between conditions. (A) Mean signal amplitude, defined via parameter estimates in a GLM, were obtained for each participants and averaged for all voxels in the key ROI from Figure 3b, early visual cortex (defined as the union of V1 and V2). No significant difference was observed (paired t-test, $t_{34} = -0.57$, $p = 0.57$, $d = 0.10$; Bayesian paired t-test, BF10 = 0.21). Grey dots represent single participants, lines represent within-participant differences, white dots, boxes and whiskers represent between-participant medians, quartiles and 1.5 interquartile ranges, respectively. **(b)** Same as in **(a)** but displaying the pairwise differences only.

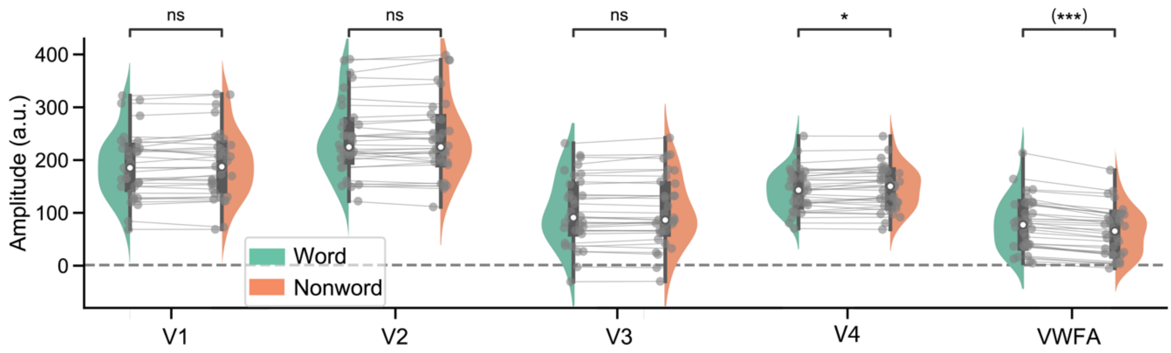

Supplementary Figure 5. Univariate results for various ROIs. Same as Supplementary Figure 4 but for 4 anatomically defined visual regions (V1-V4) and one functionally defined region (VWFA). Overall, there were no strong amplitude differences between conditions in most regions of interest, except for VWFA where BOLD amplitude was by definition higher for words than nonwords in each subject. Significance levels: * indicates $p<0.05$ (uncorrected), and (***) indicates difference-by-definition (no stats).

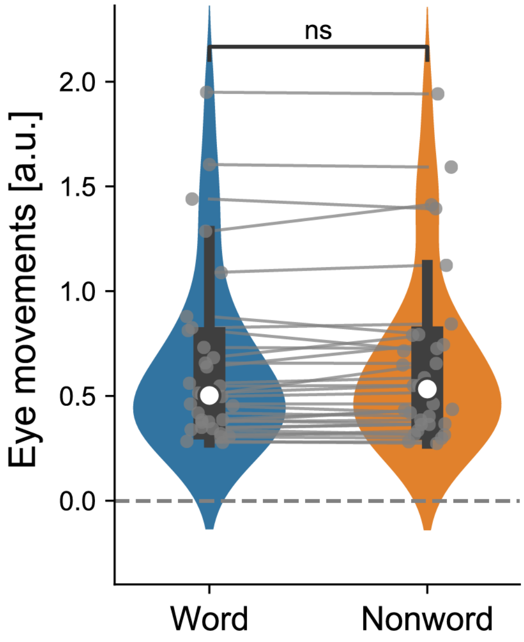

Supplementary Figure 6. Comparison of reading-related eye movements across conditions. Horizontal eye movements were quantified for each trial and then averaged for both conditions and compared within participants. Grey dots and connecting lines represent single participants, white dots group medians, boxes and whiskers represent quartiles and 1.5 interquartile ranges. No statistically significant difference between conditions was found (paired t-test, t_{32}=-1.43 P=0.16). Two participants were not included because there was no eye tracking data of sufficient quality.

Supplementary Note 1

Spatial and retinotopic specificity

If the letter information extracted from visual cortex, and its enhancement by word contexts, indeed reflect sensory representations, then the MVPA results should be retinotopically specific. If, on the other hand, letter identity could be decoded from voxels throughout much of the brain, or if the enhancement was not retinotopically specific (e.g. reflecting a more general increase in signal-to-noise ratio) it would be more difficult to conclude that the MVPA results reflect sensory representations. We therefore tested for spatial specificity by running a searchlight version of the classification and pattern correlation analyses. **Supplementary Figure 7** and **Supplementary Figure 8** depict the group averaged results of both analyses. In both figures, the *colour* of the overlay represents the difference in letter decoding between conditions (word minus nonword), while the *opacity* represents the extent to which the overall letter decoding is above chance (irrespective of condition). This way, the difference between conditions is only visible when the overall decoding was above chance. From **Supplementary Figure 7** and **Supplementary Figure 8**, two things become clear. First, opacity is nonzero almost exclusively in visual regions, implying that only there decoding was above chance, and that the letter decoding was could not have relied on a global pattern, but only on information from visual cortex. Second, most of the overlay is red. This means that in the regions with above-chance decoding, the difference between conditions is almost always positive. This converges with **Supplementary Figure 3**, by confirming that this pattern of effects was not contingent on the specific (but arbitrary) ROI definition we employ.

Supplementary Figure 7 and **Supplementary Figure 8** clearly show that letter decoding is specific to visual cortex. However, from the maps it is difficult to see if, *within* visual cortex, the letter decoding and representational enhancement peak the expected (foveal) location. This is because the individual maps got smeared out during averaging in standard space. Therefore, we ran a more sensitive ROI analysis in native EPI space. Here, we use the resulting searchlight maps (containing classification and pattern correlation results for each voxel in a participant's native EPI space). We compared the classification in the central ROI (using the functional definition described earlier) to a functionally defined peripheral ROI. Voxels were deemed peripheral when they showed a strong response to stimuli in the main experiment (which spanned a large part of the visual field), but showed a weak or no response to stimuli in the localiser (which were presented near fixation). For this analysis we focused on V1, because it has the strongest retinotopy. Indeed, as can be seen in **Supplementary**

Figure 9, overall letter decoding was greatly reduced for the peripheral ROI compared to the central ROI, both for the classification analysis (paired t-test, t_{34}=15.59, p = 8.86 × 10^{-17}, d = 2.67) and pattern correlation analysis (paired t-test, t_{34}=8.06, p = 2.65 × 10^{-9}, d = 1.38). Critically, a similar reduction in the peripheral ROI was found for the enhancement effect (the difference in decoding between conditions), again both for the classification analysis (paired t-test, t_{34}=2.56, p = 0.015, d = 0.44) and pattern correlation analysis (paired t-test, t_{34}=2.92, p = 6.31 × 10^{-3}, d = 0.50). Importantly, although we initially (**Supplementary Figure 9**) focussed on V1 – because it has the strongest retinotopy and because it was requested by the reviewer – a similar reduction was observed for our main ROI of interest, early visual cortex (i.e. the conjunction of V1 and V2). Specifically, here too we found greatly reduced overall letter decoding, both for the classification analysis (paired t-test, t_{34}=18.49, p = 5.52 × 10^{-19}, d = 3.17) and pattern correlation analysis (paired t-test, t_{34}=8.86, p = 3.02 × 10^{-10}, d = 1.52). Moreover, we again found a reduction of the enhancement effect, again both for the classification analysis (paired t-test, t_{34}=2.44, p = 0.02, d = 0.42) and pattern correlation analysis (paired t-test, t_{34}=3.21, p = 2.90 × 10^{-3}, d = 0.55). Together, these analyses show that MVPA results exhibit spatial and retinotopic sensitivity, which suggests that the MVPA results indeed reflect early visual representations, as expressed in BOLD activity.

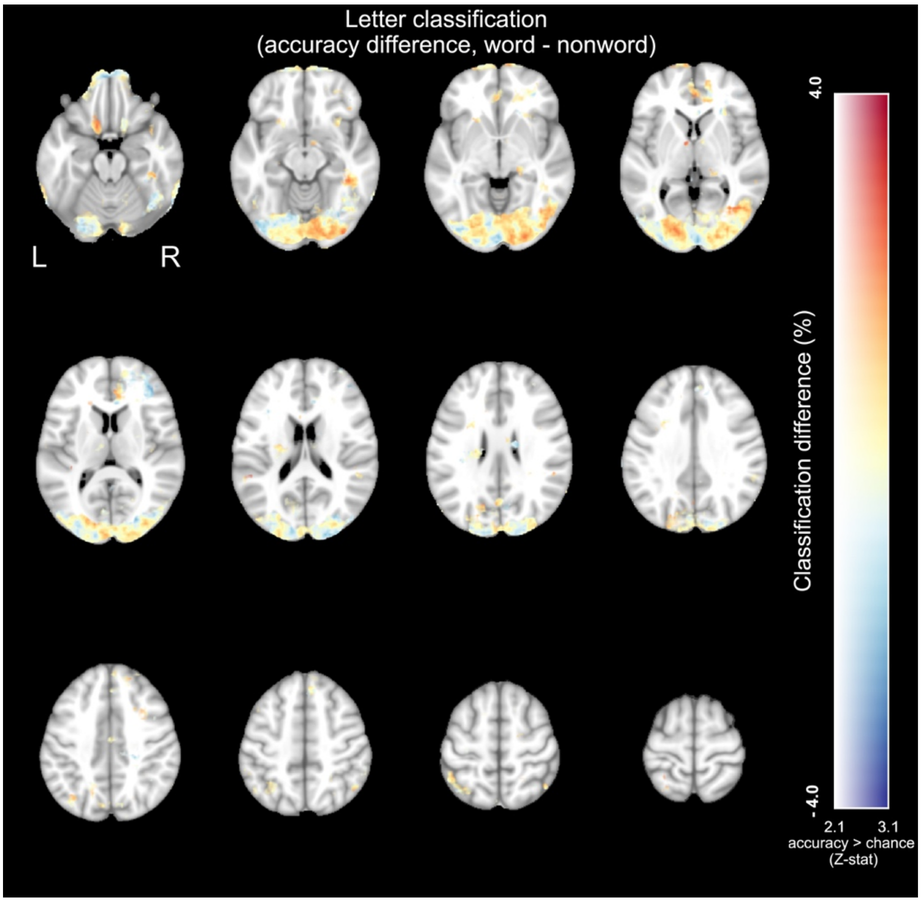

Supplementary Figure 7. **Spatial specificity of classification analysis**. Group averaged result of the searchlight version of the classification analysis. This figure uses a dual-coding scheme in which the opacity of the overlay is determined by the average decoding accuracy with respect to chance (averaged over subjects), and the colour indicates the average decoding difference (word-nonword) between conditions. See text (Supplementary Note 1) for interpretation.

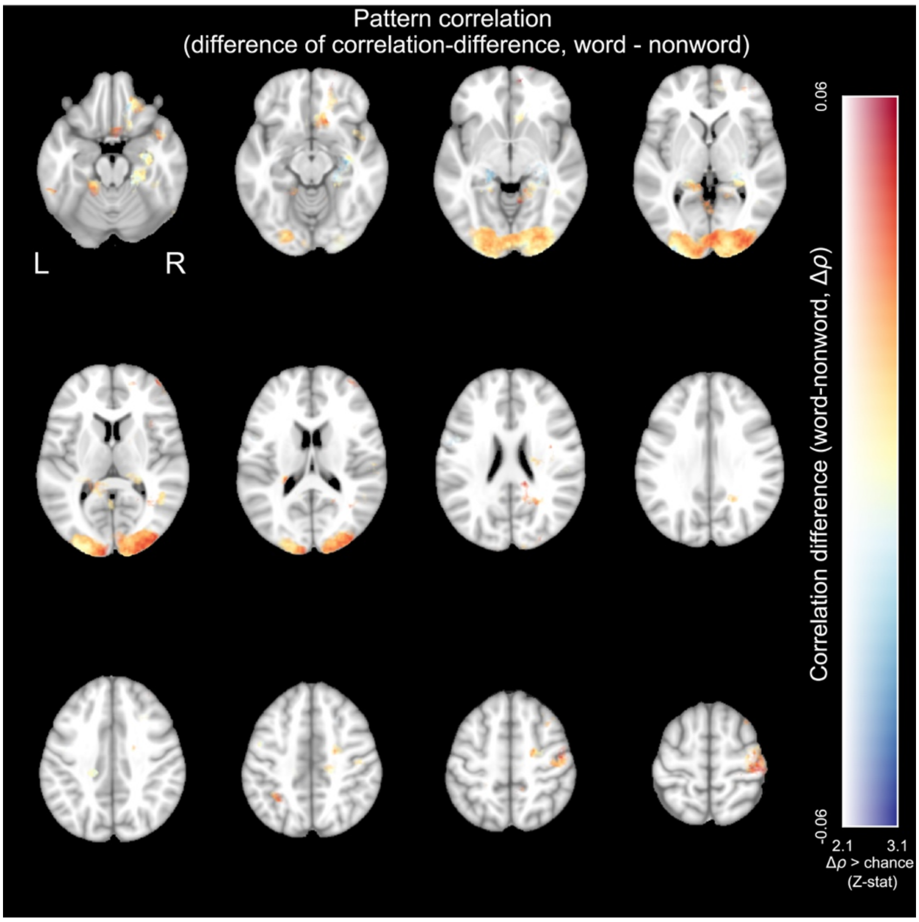

Supplementary Figure 8. Spatial specificity of pattern correlation analysis. Group averaged result of the searchlight version of the pattern correlation analysis. Results are displayed using a dual-coding scheme in which the opacity of the overlay is determined by the average letter decoding performance (quantified as pattern correlation difference) with respect to chance, and the colour indicates the decoding difference between conditions (word-nonword). See text (Supplementary Note 1) for interpretation.

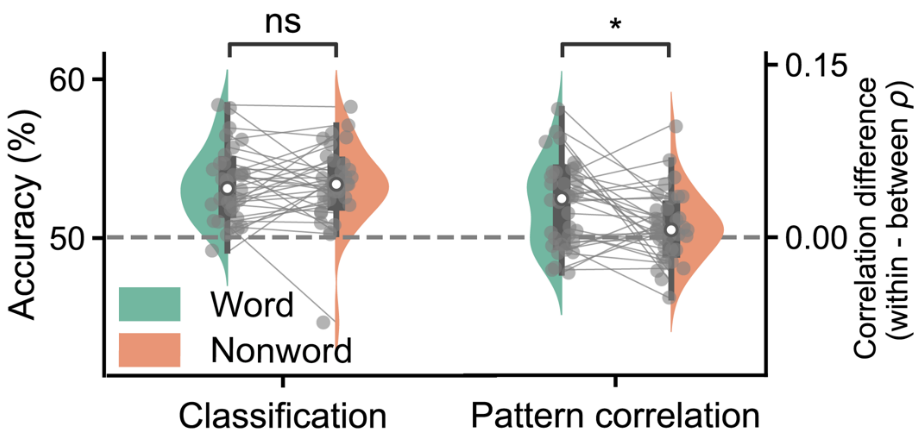

Supplementary Figure 9 Reduced letter decoding and representational enhancement in the periphery. Same analysis as in **Figure 3b**, but now for the peripheral V1 ROI (individually defined for each participant). Compared to the central V1 ROI, both classification and pattern correlation analyses revealed a reduction, both for overall letter decoding (both *p*'s < 10^{-8}, paired t-test), and representational enhancement (both *p*'s < 0.016, paired t-test). This reduction suggests both analyses relied on retinotopically specific, early sensory information. The same effect is found when this analysis is performed on early visual cortex (see text). Grey dots with connecting lines are individual participants. Colours are estimated densities, white dots are group medians, boxes are quartiles and whiskers are 1.5 interquartile range. Significance stars indicate p<0.05 (*) in a (paired) two-tailed t-test

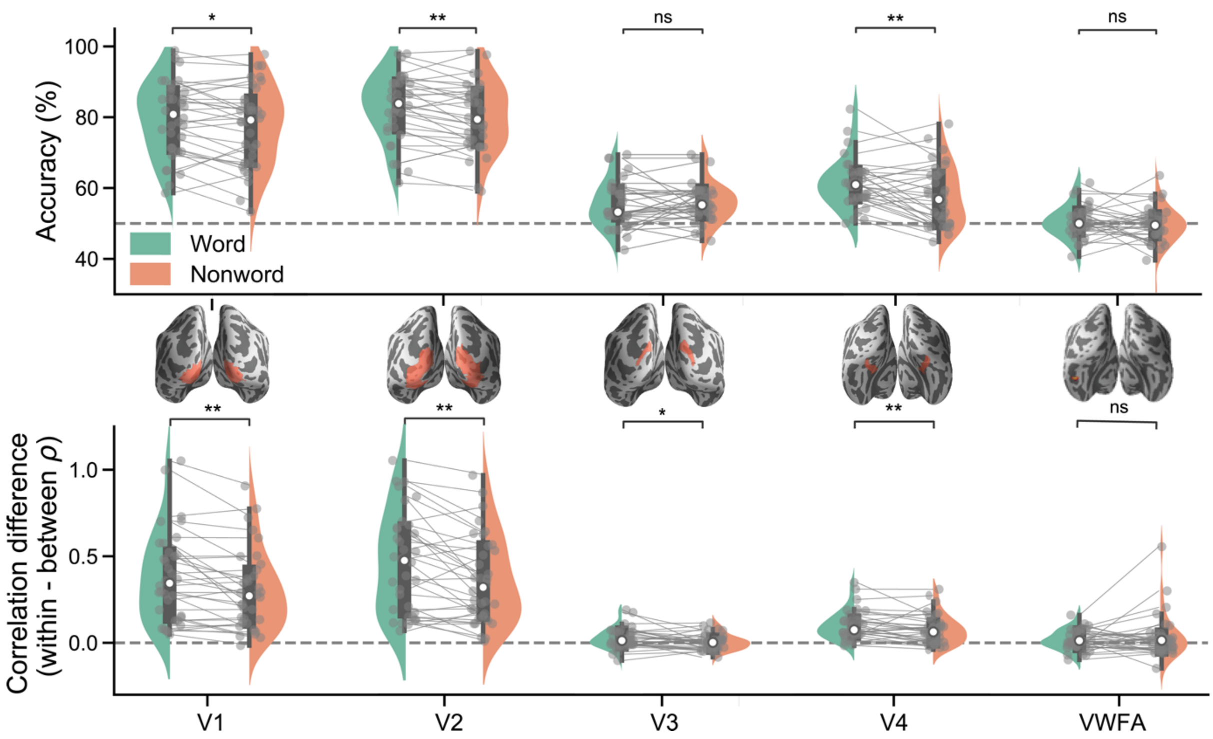

Supplementary Figure 10. Enhancement throughout the visual hierarchy. Same analysis as in Figure 3b, over the same ROIs as in Supplementary Figure 5. Overall, in all three ROIs where overall letter decoding was well-above chance, the key enhancement effect was found; in no ROI was the pattern inverted. Specifically, both classification and pattern correlation analyses revealed evidence for word enhancement in V1 (classification analysis $t_{34} = 2.35$, $P = 0.025$, d = 0.40; correlation difference: Wilcoxon signed rank $T_{34} = 115$, $P = 1.81 \times 10^{-3}$, r = 0.61) V2 (classification difference: t_{34}=3.043; P = 4.57 x 10^{-3}, d = 0.52; correlation difference: Wilcoxon's $T_{34} = 99.0$, $P = 6.90 \times 10^{-4}$, r = 0.68) and V4 (classification difference: $t_{34} = 3.42$, $p = 1.67 \times 10^{-3}$, d = 0.59; correlation difference: Wilcoxon's $T_{34} = 151.0$, $P = 0.012$, r = 0.49). However, no consistent differences were found for V3 (classification difference, Wilcoxon's $T_{34} = 176$, $P = 0.54$, r = 0.13; correlation difference: Wilcoxon's $T_{34} = 172$, $P = 0.032$, r = 0.42; see figure and note difference in direction); and VWFA (classification difference: $t_{34} = 1.18$, $p = 0.25$, d = 0.20; correlation difference: Wilcoxon's $T_{34} = 151.0$, $P = 0.012$, r = 0.49). Brain images are surface plots with anatomical ROI overlays created using the pysurfer plotting engine[2].

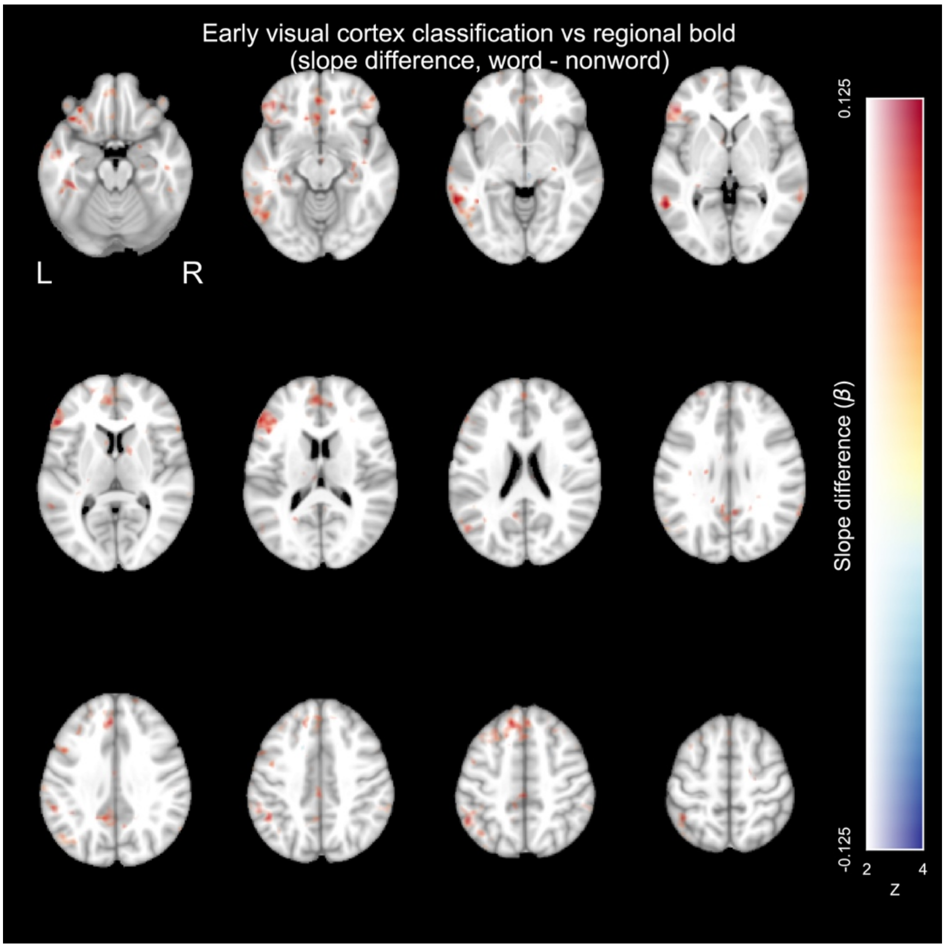

Supplementary Figure 11 Non-thresholded whole brain result of the information-activation coupling analysis. Same results as in **Figure 4c,** but using a dual coding scheme in which the overlay is opacity-weighted by statistical values instead of a binarily thresholded at statistical significance. Colour indicates the numerical difference in the information activation coupling parameter between conditions (word-nonword), opacity represents the consistency of this difference over participants, expressed using the Z-statistic. From the results it becomes evident that even without thresholding, the lateralisation, and two statistically significant clusters in left MTG and IFG, clearly stand out.

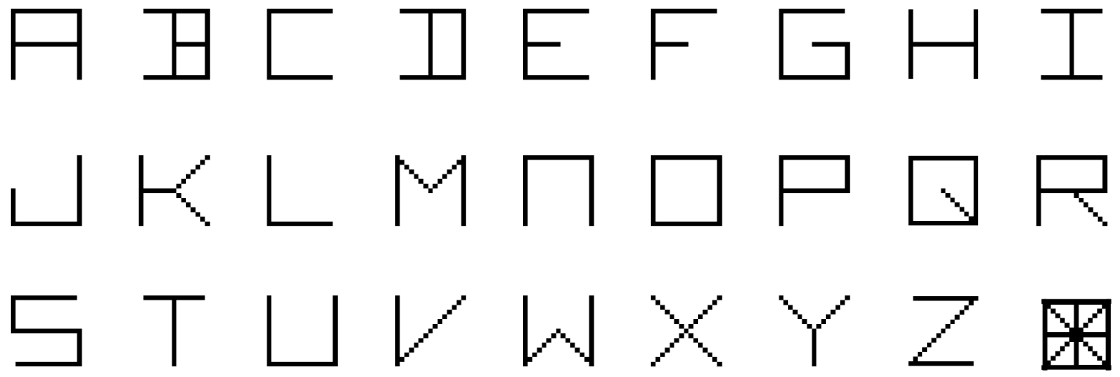

Supplementary Figure 12 Illustration of virtual font. Illustration of the virtual font presented to the network. In this font all 36 alphanumeric characters can be formed from only 14 line segments. This allows each character to be encoded as a 14-dimensional input vector representing visual features. Font is adapted from Rumelhart and Siple[3], slightly modified to increase similarity between U and N, and overlap with other letters, as we used in our experiment.

U_{word}		$U_{nonword}$		N_{word}		$N_{nonword}$	
ABUIS	KRUIK	REUJZ	AEUEI	AGNES	LYNCH	NMNNS	DSNEN
ACUTE	KRUIP	KNUUE	IOUST	BANDS	MANDY	DTNAI	INNTE
ACUUT	KRUIS	DGUNE	RNUAH	BANEN	MANEN	ILNTN	IENNW
AZUUR	KRUIT	ITUOD	DGUWD	BANGE	MENEN	HSNND	MTNSA
BEURS	LAUDE	TGUAE	OEUAT	BANJO	MENGT	NKNSE	NTNBW
BEURT	LEUKE	LNUOT	ASUEA	BANKS	MENIG	AINKH	ETNKD
BLUES	LEUKS	EDUTB	ENUDP	BENDE	MINST	JDNIV	VJNTS
BLUFT	LEUNT	NIUDL	TKUEP	BENEN	MINUS	ARNWT	ITNEI
BOUWT	MEUTE	ONUHB	OAUPI	BENUL	MONTE	IHNTR	MDNJT
BRUID	MOUTH	NPUAO	JNUCE	BINDT	NANNY	LONRH	ERNLM
BRUIN	NAUWE	FDUDE	DHUJD	BINGO	NINJA	NKNWV	DTNCA
BRULT	NEURO	EIUSP	EDUSJ	BONEN	OPNAM	GNNRT	MUNJE
BRUTE	PAUZE	LNUME	MLUHN	BONES	PANTY	RTNBE	AONRL
BRUTO	PLUIM	AGUEK	OWUAO	BONUS	PINDA	ENNTL	KRNBG
BRUUT	PLUIS	RZUNI	MNUDV	CONGO	PUNCH	DINRD	NMNCN
BUURT	PLUKT	EAUYI	ONUIE	DANDY	RANCH	NRNMI	ZDNNH
COUPE	PRUIK	WVUGN	TDUER	DANKT	RENDE	WVNVS	NDNEA
DEUGD	PRUIM	HLUOR	NTURN	DANST	RENTE	RVNNE	MVNAM
DEUGT	RAUWE	OAUWV	ENUAW	DENKT	RONDE	RDNRA	RTNXV
DRUGS	REUMA	ITUNB	DZUEO	DINER	RUNDE	EWNDZ	DJNET
DRUIF	REUZE	AIUVS	NLURE	DONOR	SANDS	IHNOI	LHNNE
DRUKT	ROUGE	RHUEJ	JDUNE	DONUT	SENOR	TPNLK	AJNCN
DRUMS	ROUTE	EHUDB	EBUUI	DUNNE	SINDS	ZTNZE	TNNSE
DUURT	ROUWT	IEUOI	NMURF	EINDE	SONAR	ZGNRE	NLNUI
EEUWS	SAUNA	NHUEZ	WVUNI	FONDS	SONDE	KDNNA	DRNLZ
ERUIT	SLUIP	DEUEO	NUUDA	GENAS	SONGS	ENNRH	DLNEN
FAUNA	SLUIS	AEUVR	SUUET	GENEN	TANGO	DRNEG	NCNEH
FLUIT	SLUIT	ZKUEN	EOUUN	GENIE	TANKS	VNNAE	CNNWI
FOUTE	SLURF	FWUTE	TAULR	GENOT	TANTE	IENWR	ARNNK
FOUTS	SLUWE	SBUAI	EIUAW	GENRE	TENEN	EINAT	RDNMH
FRUIT	SNUIF	AEUVO	NMUEN	GINDS	TONEN	JVNNR	JDNNS
GEUIT	SNUIT	HRUEN	TKUES	GUNST	TONIC	OCNEO	EDNRG
GOUWE	SNURK	GHUOW	VAUAO	HANGT	VANAF	FZNND	NMNTP
HEUSE	SPUIT	TLULZ	UNUEA	HINTS	VANGT	TBNRK	EONNI
HOUDT	SPUUG	EAUAG	VTUNL	INNEN	VENUS	PTNVO	WTNHE
HOUSE	SPUWT	EVUNE	EIUOA	JONGE	VINDT	KSNGI	DTNWT
HUURT	SQUAD	RHUET	ZMUVT	KANON	WENEN	THNLR	DLNTE
JEUGD	STUFF	NPUAL	THUIJ	KENDE	WENST	UONTD	DRNAE
JEUKT	STUIT	JHUTZ	ETUDL	KINDS	WINDT	TNNRI	RDNZJ
JOUWE	STUKS	TXUEM	VHUOR	KINKY	WINST	DRNNM	SINNO
KAUWT	STUNT	HRUMN	EAUAR	KUNST	WONDE	MNNGO	OTNUE
KEURT	STUUR	NIUEH	AUULS	LANDT	WONEN	GRNEM	WLNEH
KEUZE	THUIS	NTUEL	OGUBN	LANGE	ZENDT	DTNJI	VWNOE
KLUIF	TRUCK	TGURV	ANUET	LANGS	ZENUW	EANAC	TLNEG
KLUIS	TRUCS	HDUPM	NLUAR	LENEN	ZINGT	IENNG	EONNA
KLUNS	TRUST	MNUHC	ODUAL	LENTE	ZINKT	ETNIR	NDNTN
KLUTS	TRUUK	DLUEI	EHUWJ	LINIE	ZONDE	TZNRO	TDNLT
KOUDE	VUURT	VNUDW	PEUEA	LINKS	ZONEN	EMNSC	IHNSE
KOUDS	ZEURT	ZUUAH	TNUEV	LONEN	ZONES	IGNEM	ODNRB
KRUID	ZOUTE	HGUTO	ENUIZ	LUNCH	ZONET	VNNAR	KMNHT

Supplementary Table 1. Word and nonword stimuli used in main experiment. Words were taken from a corpus scraped from a large number of subtitles and hence also contains names and common English terms that are not Dutch words in a strict sense. However, all word items are familiar and pronounceable, whereas all nonword items are unfamiliar and unpronounceable.

Supplementary references

1. Richter, D., Ekman, M. & Lange, F. P. de. Suppressed Sensory Response to Predictable Object Stimuli throughout the Ventral Visual Stream. *J. Neurosci.* **38**, 7452–7461 (2018).
2. Ramachandran, P. & Varoquaux, G. Mayavi: 3D visualization of scientific data. *Comput. Sci. Eng.* **13**, 40–51 (2011).
3. Rumelhart, D. E. & Siple, P. Process of recognizing tachistoscopically presented words. *Psychol. Rev.* **81**, 99–118 (1974).
