# Supplementary material for: Word contexts enhance the neural representation of individual letters in early visual cortex
Source: Nat Commun. 2020 Jan 16;11:321. doi: 10.1038/s41467-019-13996-4 (PMC6965097; doi:10.1038/s41467-019-13996-4)
Supplement: Supplementary file 3 — Reporting Summary [file 41467_2019_13996_MOESM3_ESM.pdf]

nature research

Corresponding author(s): Micha Heilbron, Floris de Lange

Last updated by author(s): Jul 28, 2019

Reporting Summary

Nature Research wishes to improve the reproducibility of the work that we publish. This form provides structure for consistency and transparency in reporting. For further information on Nature Research policies, see Authors & Referees and the Editorial Policy Checklist .

Statistics

For all statistical analyses, confirm that the following items are present in the figure legend, table legend, main text, or Methods section.

n/a	Confirmed	
☐	☒	The exact sample size (*n*) for each experimental group/condition, given as a discrete number and unit of measurement
☐	☒	A statement on whether measurements were taken from distinct samples or whether the same sample was measured repeatedly
☐	☒	The statistical test(s) used AND whether they are one- or two-sided<br>*Only common tests should be described solely by name; describe more complex techniques in the Methods section.*
☐	☒	A description of all covariates tested
☐	☒	A description of any assumptions or corrections, such as tests of normality and adjustment for multiple comparisons
☐	☒	A full description of the statistical parameters including central tendency (e.g. means) or other basic estimates (e.g. regression coefficient) AND variation (e.g. standard deviation) or associated estimates of uncertainty (e.g. confidence intervals)
☐	☒	For null hypothesis testing, the test statistic (e.g. *F*, *t*, *r*) with confidence intervals, effect sizes, degrees of freedom and *P* value noted<br>*Give P values as exact values whenever suitable.*
☐	☒	For Bayesian analysis, information on the choice of priors and Markov chain Monte Carlo settings
☒	☐	For hierarchical and complex designs, identification of the appropriate level for tests and full reporting of outcomes
☐	☒	Estimates of effect sizes (e.g. Cohen's *d*, Pearson's *r*), indicating how they were calculated

Our web collection on statistics for biologists contains articles on many of the points above.

Software and code

Policy information about availability of computer code

Data collection	psychtoolbox-3
Data analysis	fsl 5.0.11<br>sklearn 0.2<br>nilearn 0.5.0<br>nistats 0.0.1b<br>python 3.5<br>anaconda3<br>MATLAB 2017b

For manuscripts utilizing custom algorithms or software that are central to the research but not yet described in published literature, software must be made available to editors/reviewers. We strongly encourage code deposition in a community repository (e.g. GitHub). See the Nature Research guidelines for submitting code & software for further information.

Data

Policy information about availability of data

All manuscripts must include a data availability statement. This statement should provide the following information, where applicable:

- Accession codes, unique identifiers, or web links for publicly available datasets
- A list of figures that have associated raw data
- A description of any restrictions on data availability

All raw data and custom code to reproduce all analyses and recreate each figure is published in the Donders Data Repository. Temporary (reviewer) link is found in the manuscript. Permanent (public) link will be found in the paper upon publication.

Field-specific reporting

Please select the one below that is the best fit for your research. If you are not sure, read the appropriate sections before making your selection.

☒ Life sciences ☐ Behavioural & social sciences ☐ Ecological, evolutionary & environmental sciences

For a reference copy of the document with all sections, see nature.com/documents/nr-reporting-summary-flat.pdf

Life sciences study design

All studies must disclose on these points even when the disclosure is negative.

Sample size	Sample size was chosen to detect a within-subject effect of at least medium size (d>0.5) with 80% power using a two-tailed one-sample (or paired) t-test.
Data exclusions	We used two pre-defined inclusion criteria. First, baseline letter classification accuracy (averaged over both conditions) had to be significantly above chance. This lead to the exclusion of one participant, who had moved excessively between runs. Second, participants were not allowed to have their eyes shut for an extended duration in more than 25 trials. This led to the exclusion of another participant, which had many trials during which the eyes fell shut. As such, we in total excluded 2 out of the 36 original participants (±5%). Importantly, excluding the participants did not change any of the conclusions of any of the key tests.
Replication	While we have not performed a direct replication of the current study, the following measures to ensure replicability of the reported results. First, all key analyses concern a-priori defined, theoretically driven comparisons. Second, we chose sample size of 34 to ensure we could detect a within-subject effect of at least medium size (d>0.5) with 80% power. All key results were indeed in that effect-size range (i.e. >0.5) and well below our predefined threshold of statistical significance (alpha=0.05, highest p-value of key comparisons: p=7.55×10-3). Finally, we have shared all raw data and computer code to reproduce and inspect the analyses leading to the results reported here.
Randomization	All analyses rely on conditions that are manipulated within subjects (not between groups).
Blinding	All analyses rely on conditions that are manipulated within subjects (not between groups).

Reporting for specific materials, systems and methods

We require information from authors about some types of materials, experimental systems and methods used in many studies. Here, indicate whether each material, system or method listed is relevant to your study. If you are not sure if a list item applies to your research, read the appropriate section before selecting a response.

Materials & experimental systems

n/a	Involved in the study	
☒	☐	Antibodies
☒	☐	Eukaryotic cell lines
☒	☐	Palaeontology
☒	☐	Animals and other organisms
☐	☒	Human research participants
☒	☐	Clinical data

Methods

n/a	Involved in the study	
☒	☐	ChIP-seq
☒	☐	Flow cytometry
☐	☒	MRI-based neuroimaging

Human research participants

Policy information about studies involving human research participants

Population characteristics	We used normal healthy participants that were selected via report to a public advertisement in the participant pool from the Donders Institute. Of all participants, the mean ±SD age was 23 ± 3.32, and 12 of 34 were male.
Recruitment	Participants were recruited from the pool of experimental participants at the Donders Institute, and enrolled as they answered to our advertisement.
Ethics oversight	The study was in accordance with the institutional guidelines of the local ethical committee (CMO region Arnhem-Nijmegen, The Netherlands)

Note that full information on the approval of the study protocol must also be provided in the manuscript.

Magnetic resonance imaging

Experimental design

Design type	Block design
Design specifications	We used a blocked design, in which we presented blocks of four long trials (one of each of the four conditions), followed by a null-trial experimental run consisted of 40 trials of 14 s. Trials were presented in blocks consisting of 5 trials: one of each condition (U-word, U-nonword, N-word,N-nonword), plus a null trial during which only the fixation dot was present. The order of trial types within blocks was randomised and equalised: over the entire experiment, each order was presented twice, resulting in a total number of 240 trials (192 excluding nulls).
Behavioral performance measures	We assessed accuracy and reaction times. For accuracy we analysed the mean accuracy between conditions

Acquisition

Imaging type(s)	anatomical, functional
Field strength	3T
Sequence & imaging parameters	Functional images were acquired using a whole-brain T2*-weighted multiband-4 sequence (TR/TE = 1400/33.03 ms, voxel size = 2 mm isotropic, 75° flip angle, A/P phase encoding direction). Anatomical images were acquired with a T1-weighted MP-RAGE (GRAPPA acceleration factor = 2, TR/TE = 2300/3.03 ms, voxel size 1 mm isotropic, 8° flip angle).
Area of acquisition	whole brain

Diffusion MRI ☐ Used ☒ Not used

Preprocessing

Preprocessing software	FSL 5.0.11
Normalization	Most key analyses were performed in native space and therefore not spatially normalised. The only exception is the whole-brain information-activation coupling analyses, for which functional images were registered to the anatomical image using boundary based registration as implemented in FSL's FLIRT and subsequently to the MNI152 T1 2 mm template brain using linear registration with 12 degrees of freedom.
Normalization template	MNI152
Noise and artifact removal	Nuisance regressors were added for the first-order temporal derivatives of the regressors of interest (to account for HRF variability), and 24 motion regressors (6 motion parameters plus their Volterra expansion, following Friston et al., 1996).
Volume censoring	n/a

Statistical modeling & inference

Model type and settings	For the key analyses, we used multivariate analysis in which we quantified representational fidelity for both word and nonword conditions and compared these within-participants
Effect(s) tested	For univariate, multivariate, and coupling analysis, we compared word to nonword conditions in a pair-wise fashion, using a paired t-test or its non-parametric alternative: the Wilcoxon Sign Rank test.

Specify type of analysis: ☐ Whole brain ☐ ROI-based ☒ Both

Anatomical location(s)	We used a combined functional/anatomical approach to ROI definition, in which we first defined a broad anatomical ROI, and then within that ROI selected a limited number of voxels based on a functional contrast orthogonal to the comparison of interest. Specifically, anatomical ROIs were based on a subject-specific cortical parcellation (mainly the Desikan-Killiany atlas) via Freesurfer. The main voxel selection comparison was stimulus>baseline during the letter localiser experiment (see paper for details).
Statistic type for inference (See Eklund et al. 2016)	Cluster-wise using the default cluster-forming threshold of $z > 3.1$ (i.e., $p < 0.001$) and a cluster significance threshold of $p < 0.05$.
Correction	We used FSL's Gaussian random-field based cluster thresholding, using the default cluster-forming threshold of $z > 3.1$ (i.e., $p < 0.001$) and a cluster significance threshold of $p < 0.05$.

Models & analysis

n/a	Involved in the study
☐	☒ Functional and/or effective connectivity
☒	☐ Graph analysis
☐	☒ Multivariate modeling or predictive analysis

Functional and/or effective connectivity	We used a GLM-based PPI-like analysis in the information-activation coupling analysis. Here, letter information (classifier probability) in early visual cortex was modelled as a linear function of BOLD activation in orthography-sensitive areas like VWFA, and we tested for an increase in slope in word compared to nonword conditions. To correct for temporal autocorrelation, pre-whitening was applied before model estimation, using an AR(1) autoregressive noise model implemented in nistats.
Multivariate modeling and predictive analysis	For the classification analysis we used a logistic regression classifier implemented in in sklearn 0.2 with all default settings. The model was trained on the time-averaged data from the functional localiser run and tested on the time-averaged data from the experimental runs. Because we had the same number of samples for each class, binary classification performance was evaluated using accuracy (%).<br><br>For both training and testing, we used spatially non-smoothed, motion-corrected, high-pass filtered (128s) data. Data were temporally filtered using a third-order Savitzky-Golay low-pass filter (window length 21) and *z*-scored for each run separately
